# Improved Antitumor Effect of NK Cells Activated by Neutrophils in a Bone Marrow Transplant Model

**DOI:** 10.1155/2023/6316581

**Published:** 2023-01-31

**Authors:** Daisuke Nakato, Shotaro Iwamoto, Keishiro Amano, Takahiro Ito, Hidemi Toyoda, Ryo Hanaki, Mari Morimoto, Kaori Niwa, Isao Tawara, Kyoko Imanaka-Yoshida, Masahiro Ogawa, Masahiro Hirayama

**Affiliations:** ^1^Department of Pediatrics, Mie University Graduate School of Medicine, Tsu, Mie, Japan; ^2^Department of Hematology and Oncology, Mie University Graduate School of Medicine, Tsu, Mie, Japan; ^3^Department of Pathology and Matrix Biology, Mie University Graduate school of Medicine, Tsu, Mie, Japan; ^4^Department of Pediatrics, National Hospital Organization Mie-Chuo Medical Center, 2158-5, Hisaimyojincho Hisai, Tsu, Mie, Japan

## Abstract

The licensing process mediated by inhibitory receptors of the Ly49 C-type lectin superfamily that recognizes self-major histocompatibility complex (MHC) class I in mice is essential for the proper antitumor function of natural killer (NK) cells. Several models for NK cell licensing can be exploited for adoptive immunotherapy for cancer. However, the appropriate adoptive transfer setting to induce efficient graft versus tumor/leukemia effects remains elusive, especially after hematopoietic stem cell transplantation (HSCT). In our previous experiment, we showed that intraperitoneal neutrophil administration with their corresponding NK receptor ligand-activated NK cells using congenic mice without HSCT. In this experiment, we demonstrate enhanced antitumor effects of licensed NK cells induced by weekly intraperitoneal injections of irradiated neutrophil-enriched peripheral blood mononuclear cells (PBMNCs) in recipient mice bearing lymphoma. Bone marrow transplantation was performed using BALB/c mice (H-2^d^) as the recipient and B10 mice (H-2^b^) as the donor. The tumor was A20, a BALB/c-derived lymphoma cell line, which was injected subcutaneously into the recipient at the same time as the HSCT. Acute graft versus host disease was not exacerbated in this murine MHC class I mismatched HSCT setting. The intraperitoneal injection of PBMNCs activated a transient licensing of NK subsets expressed Ly49G2, its corresponding NK receptor ligand to H-2^d^, and reduced A20 tumor growth in the recipient after HSCT. Pathological examination revealed that increased donor-oriented NK1.1+NK cells migrated into the recipient tumors, depending on neutrophil counts in the administered PBMNCs. Collectively, our data reveal a pivotal role of neutrophils in promoting NK cell effector functions and adoptive immunotherapy for cancer.

## 1. Introduction

Hematopoietic stem cell transplantation (HSCT) from allogeneic donors provides a curative therapy for patients with malignant disease [1]. Although disease relapse is a major obstacle to successful HSCT in some patients, HSCT may potentially benefit from the graft-versus-leukemia/tumor (GVL/GVT) effects [2, 3]. Among the effectors of GVL/GVT, alloreactive natural killer (NK) cells are increasingly being recognized as an important component of the overall antileukemia and antitumor effects [3, 4].

NK cells physiologically modulate the immune system and mediate the direct killing of malignant tumors or infected cells [5]. Moreover, NK cells may recognize the missing self, i.e., the lack of normal expression of major histocompatibility complex (MHC) class *Ι* molecule [6]. NK cells require the engagement of the inhibitory receptor with MHC class *Ι* to attain functional competence. This process termed licensing (education) allows NK cells to be activated through activation receptors; activated NK cells can detect and kill cells lacking self-MHC class I [6]. Murine NK cells express inhibitory receptors of the Ly49 C-type lectin superfamily interacting with H-2, one of the classic MHC class I antigens.

The mechanisms of NK cell maturation are gradually being elucidated. NK cell licensing may be caused by the presentation of self-MHC class I derived from hematopoietic cells and lymphoid organs (nonhematopoietic cells) [7–9]. Intriguingly, Jaeger et al. reported that NK cell maturation occurs not only in the bone marrow but also in the lymph nodes and spleen through the interactions of NK cells with neutrophils [10]. We also demonstrated that murine NK cells were effectively activated by intraperitoneal injection of neutrophil-enriched peripheral blood mononuclear cells (PBMNCs) with corresponding NK cell receptors using a congenic murine model [11].

Here, we investigated the posttransplant transfer of neutrophils in MHC class I mismatched murine HSCT to determine if these cells provided GVL/GVT effects via neutrophil-induced activation of allogeneic NK cells. HSCT was performed using BALB/c mice (H-2^d^) as the recipient and B10 mice (H-2^b^) as the donor. In this murine HSCT model, the ligand of Ly49C/I receptor and Ly49G2 receptor of NK cells is H-2^b^ and H-2^d^, respectively [12–14]. Interestingly, intraperitoneal injection of neutrophil-enriched PBMNCs mobilized with murine G-CSF after murine allogeneic HSCT enhanced the activity of Ly49G2+NK cells and induced GVT effects in recipient mice bearing lymphoma without exacerbating graft versus host disease (GVHD). The neutrophil-enriched PBMNCs might be associated with a significant antitumor response involving NK cell licensing caused by the presentation of self MHC class I derived from recipient nonhematopoietic cells; thus, adoptive immunotherapy with neutrophils, which activate licensed NK cells, is expected to be useful for human HSCT.

## 2. Material and Methods

### 2.1. Animals

C57BL/10 (B10) and BALB/c female mice were purchased from Japan SLC (Shizuoka, Japan). These mice, aged 8-12 weeks, were used for all experiments. The care and breeding of animals were in accordance with institutional guidelines. All procedures used in this research were approved by the Ethical Committee (permission number 2019-17), Mie University Graduate School of Medicine.

### 2.2. Cell Line

A20 is a B-cell leukemia/lymphoma cell line of BALB/c origin that occurred spontaneously in a 15-month-old mouse. The cells are nonimmunogenic in a syngeneic host. A20 cells were continuously maintained in a culture medium consisting of RPMI 1640 supplemented with 10% heat-inactivated fetal bovine serum at 37°C and 5% CO_2_. The A20 cell line was purchased from American Type Culture Collection.

### 2.3. Antibodies and Flow Cytometry

PBMNCs suspended in phosphate-buffered saline (PBS) preincubated with Fc*γ*R blocking antibody (anti-mouse CD16/CD32) were incubated with fluorescein isothiocyanate-, phycoerythrin-, peridinin-chlorophyll protein cyanine5.5-, or allophycocyanin-conjugated monoclonal antibodies for 20 min at 4°C. Antibodies against Ly49C/I (5E6), Ly49G2 (4D11), CD11b (M1/70), CD107a (1D4B), CD49b (DX5), CD3 (17A2), H-2d (34-1-25), H-2b (28-8-6), and Ly-6G/Ly-6C (Gr-1) (RB6-8C5) were purchased from BD Bioscience, BioLegend, and eBioscience. After staining, the cells were washed twice with PBS, incubated with propidium iodide at room temperature for 5 min, and subjected to fluorescence-activated cell sorting (FACS). Activated NK cells were gated as CD3^−^CD49b^+^CD107a^+^. Neutrophils were gated as Ly-6G/Ly-6C (Gr-1)^+^CD11b^+^. Flow cytometry was performed on a FACS Canto II using Diva software (Becton Dickinson, Franklin Lakes, NJ, USA).

### 2.4. Bone Marrow Transplantation

Mice underwent allogeneic bone marrow transplantation (BMT). Briefly, recipient BALB/c mice (H-2^d^) received 7.5 Gy total body irradiation. On day 0, recipients received a single injection of BM cells (5 × 10^6^) obtained from donor B10 mice (H-2^b^) through the tail vein within 24 h after total body irradiation.

### 2.5. Induction of NK Cell Licensing

For *in vivo* induction of NK cell licensing by neutrophils using the BMT model, 40 Gy-irradiated PBMNCs (2 × 10^6^) were collected from donor (B10) mice untreated or treated with recombinant murine granulocyte colony-stimulating factor (rmG-CSF, BioLegend, San Diego, CA) (subcutaneously, 250 *μ*g/kg/day, 5 days) [11, 15]. The PBMNCs were injected intraperitoneally into recipient BALB/c mice on day 21 after BMT. We analyzed the expression of CD107a on Ly49C/I- (its ligand is H-2^b^) positive or Ly49G2- (its ligand is H-2^d^) positive NK cells in peripheral blood from recipient BALB/c mice before intraperitoneal injection (day 21) and 2 days (day 23) and 14 days (day 35) after intraperitoneal injection of PBS or PBMNCs to evaluate the licensing effect [11, 16, 17]. PBMNCs were isolated using red blood cell lysing buffer (BD Biosciences, San Jose, CA).

### 2.6. Evaluation of Antitumor Effects and Acute GVHD

Recipient BALB/c mice received A20 cells (5 × 10^6^) subcutaneously on day 0 and irradiated (40 Gy) PBMNCs (prepared as described above) on days 7, 14, and 21 after BMT. The antitumor effects were evaluated by tumor size in recipient mice on days 7, 14, 21, and 28, as described previously [18]. The degree of clinical acute GVHD in recipient mice was monitored weekly for 4 weeks after BMT using a scoring system as described previously [19].

### 2.7. Histological Examination

A20 cells (5 × 10^5^) were subcutaneously injected into recipient BALB/c mice on the day of BMT (day 0). PBS or PBMNCs (40 Gy-irradiated, 2 × 10^6^) from untreated or rmG-CSF treated-B10 mice were intraperitoneally injected on day 21 after BMT. Two days after injection, the tumor engrafted in recipients was removed and fixed in 10% formalin solution, dehydrated, and embedded in paraffin. Thin-sliced sections were stained with anti-mouse NK1.1 antibody and anti-mouse CD3 antibody. Slides were examined systematically by three of the authors (D.N., S.I., and K.I.).

### 2.8. Statistical Analysis

One-way ANOVA was used to compare the differences among groups. Differences were considered statistically significant when the *p* value was <0.05.

## 3. Results

### 3.1. Distribution of Ly49 Receptors and Licensing of NK Cells in Recipient BALB/c Mice after BMT

**Figure 1:**
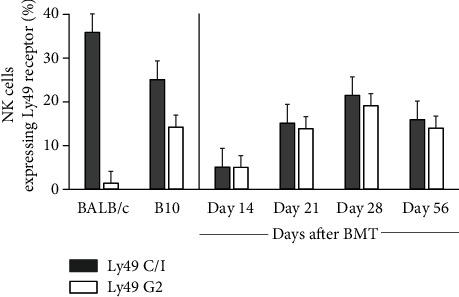
Distribution of Ly49 receptors on NK cells in recipient BALB/c mice before and after bone marrow transplantation. The distribution of Ly49 C/I^+^ and L49 G2^+^ NK cells in BALB/c mice (*n* = 12) and B10 mice (*n* = 12) before transplantation was measured. Subsequently, the distribution of transplanted mice 14 (*n* = 12), 21 (*n* = 11), 28 (*n* = 11), and 56 days (*n* = 9) after BMT was measured. BMT: bone marrow transplant; NK: natural killer.

**Figure 2:**
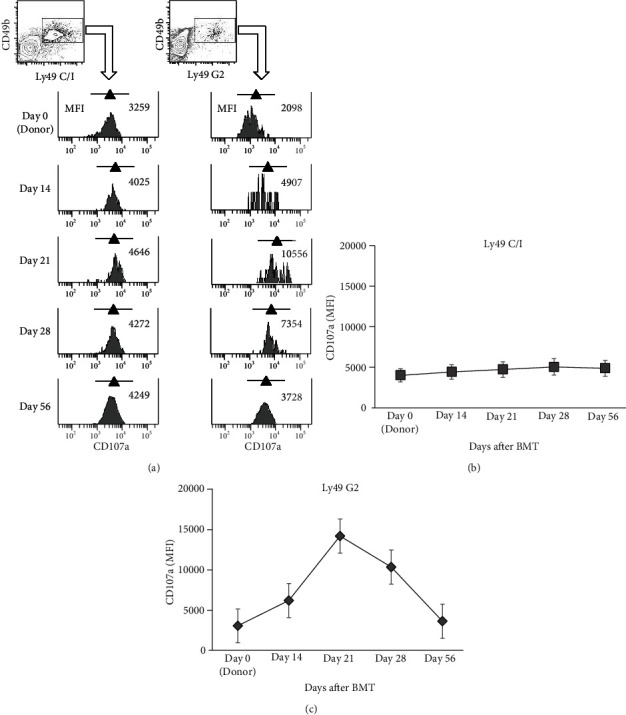
Changes in the function of NK cells after BMT. The expression of CD107a, as a functional marker for the identification of NK cell activity, was detected by flow cytometry in each Ly49C/I^+^ and Ly49G2^+^ NK cells of recipient mice before BMT (day 0: donor, *n* = 12) and on days 14 (*n* = 7), 21 (*n* = 7), 28 (*n* = 7) and 56 (*n* = 5) after BMT. The mean fluorescence intensity (MFI) is shown in number and triangle symbol in the upper right corner of each plot in the representative data (a). The activity of Ly49C/I^+^ NK cells (b) and Ly49G2^+^ NK cells (c) of recipient mice was determined as the MFI plus or minus standard deviation values of three independent experiments.

Licensing of NK cells requires Ly49 receptor family binding to MHC class I [6, 11]. Therefore, we examined the distributions of Ly49C/I and Ly49G2, which correspond to MHC class I of B10 (H-2^b^) and BALB/c (H-2^d^), respectively, in recipient BALB/c mice on the designated day after BMT [12–14]. First, full-donor chimerism was confirmed on day 14 after BMT by flow cytometric analysis of peripheral blood in recipient mice (data not shown). The percentages of Ly49C/I^+^ and Ly49G2^+^ NK cells in recipient mice peaked on day 28 and then began to decline, reaching a level comparable to NK cells in donor B10 mice on day 56 (Figure 1). There was also no significant difference in the changes in the ratios of Ly49C/I^+^ or Ly49G2^+^ NK cells until day 56. We also investigated the expression of CD107a, as a marker of NK cell functional activity, on the surface of Ly49C/I^+^ or Ly49G2^+^ NK cells of recipient mice on the same days after BMT. Flow cytometry analysis found that there was no change in the mean fluorescence intensity (MFI) of CD107a expression on Ly49C/I^+^ NK cells which mainly bind to the ligand of donor-type MHC class I, H-2^b^ (Figures 2(a) and 2(b)). On the other hand, the MFI of CD107a on Ly49G2^+^ NK cells increased and peaked on day 21 and then gradually decreased to the same level as before BMT on day 56 (Figures 2(a) and 2(c)). Surprisingly, these results indicate that NK cells expressing Ly49G2 receptors corresponding to the recipient-type MHC class *Ι* (H-2^d^) might be transiently activated via the interaction with nonhematopietic cells in recipient mice between days 21 and 28 after BMT.

### 3.2. Licensed NK Cells in Recipient Mice after BMT Enhanced by Neutrophils

**Figure 3:**
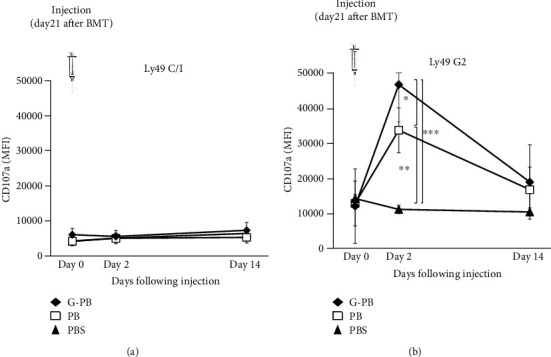
Neutrophils enhance licensed NK cells after BMT. Intraperitoneal injection of PBS (*n* = 9), PB (*n* = 9), G-PB (*n* = 5) was performed to ehnance licensed NK cell at day21 after BMT. The activation of licensed NK cells by neutrophils was measured using the MFI of CD107a of Ly49C/I^+^ NK cells (a) and Ly49G2^+^ NK cells (b) before intraperitoneal injection (day 0) and 2 days and 14 days after intraperitoneal injection. ^∗^*p* < 0.05,  ^∗∗^*p* < 0.01, and^∗∗∗^*p* < 0.001. G-PB: peripheral blood from rmG-CSF-treated mice; PB: peripheral blood from untreated B10 mice; PBS: phosphate-buffered saline.

Previously, we reported that neutrophils promote the licensing of NK cells with the corresponding NK receptor ligand in congenic mouse models such as B10 and B10.D2 [11]. We investigated whether neutrophils enhanced the licensed donor NK cells in recipient mice using a murine MHC mismatched allogeneic BMT model. We prepared PBMNCs obtained from donor-type B10 mice treated with or without rmG-CSF and injected the irradiated PBMNCs (2.0 × 10^6^) intraperitoneally in recipient BALB/c mice on day 21 after BMT. The percentage of neutrophils among the PBMNCs in peripheral blood from untreated B10 mice (PB) or rmG-CSF-treated mice (G-PB) was 12% or 70%, respectively. The licensing of NK cells by neutrophils was measured using the MFI of CD107a expression before and 2 and 14 days after intraperitoneal injection. No differences in the MFIs of CD107a expression in Ly49C/I^+^ NK cells were detected in any of the groups during this observation period (Figure 3(a)). In contrast, the activity of Ly49G2^+^ NK cells in the PB group increased significantly compared to that in the PBS group at 2 days after intraperitoneal injection (PB vs. PBS; *p* = 0.001). Moreover, the activity of Ly49G2^+^ NK cells in the G-PB group was significantly superior in comparison to that in the PBS group or the PB group at the same point (PBS vs. G-PB, *p* < 0.001; PB vs. G-PB, *p* = 0.038). However, the activity of licensed Ly49G2^+^ NK cells by neutrophils was reduced to preinjection level by 14 days after injection in all groups (Figure 3(b)).

### 3.3. Graft versus Tumor Effects of NK Cells Enhanced by Neutrophils without Exacerbating Acute GVHD

**Figure 4:**
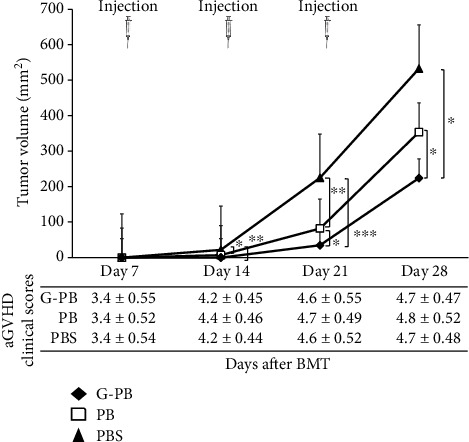
The tumor control of NK cells by neutrophils *in vivo*. The tumor was subcutaneously injected under the axilla at the same time as the BMT. PBS, PB, and G-PB were intraperitoneally injected on days 7, 14, and 21 after BMT to enhance the activity of NK cells. Tumor size measured using calipers and acute GVHD scores were checked on days 7, 14, 21, and 28. PBS: *n* = 12; PB: *n* = 10; G-PB: *n* = 10. ^∗^*p* < 0.05,  ^∗∗^*p* < 0.01, and^∗∗∗^*p* < 0.001.

Enhancement of the antitumor effects via NK cells licensing by neutrophils was confirmed in a murine MHC mismatched allogeneic BMT model. A20 cells (5 × 10^5^) that can engraft in recipient BALB/c mice were subcutaneously administered under the axilla in each donor mouse at the same time as BMT. To sustainably enhance the activity of donor NK cells after BMT, PBS, irradiated PB, or G-PB was intraperitoneally injected into recipient mice on days 7, 14, and 21 after BMT. The tumor size was measured using calipers and recorded weekly until day 28 after BMT (Figure 4).

In the PBS group, the tumor implants grew to a palpable size on day 14 and then rapidly increased. Tumor growth in the PB group was statistically suppressed compared to that in the PBS group from day 14 to day 21 (PBS vs. PB on day 14, *p* = 0.041; PBS vs. PB on day 21, *p* = 0.001; PBS vs. PB on day 28, *p* = 0.291). In the G-PB group, the tumor size was difficult to measure until day 14 (PBS vs. G-PB on day 14, *p* = 0.002; PB vs. G-PB on day 14, *p* = 0.148). Overall, tumor growth in the G-PB group was significantly suppressed in comparison to tumor sizes in the other groups during this observation period (on day 21: PBS vs. G-PB, *p* < 0.001; PB vs. G-PB, *p* = 0.012; on day 28: PBS vs. G-PB, *p* = 0.021; PB vs. G-PB, *p* = 0.049). Notably, no differences in acute GVHD clinical scores were detected between the three groups (Figure 4).

**Figure 5:**
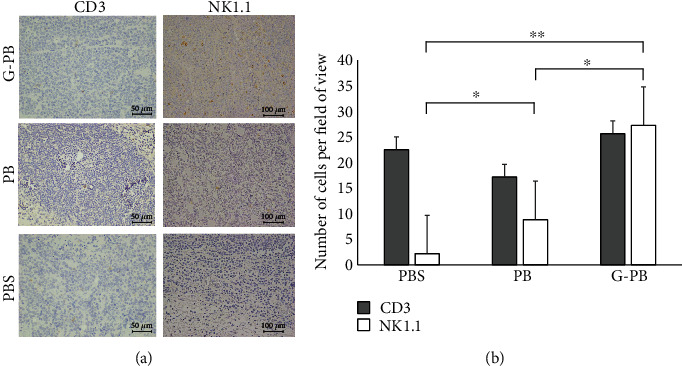
Evaluation of NK cell neutrophil-induced antitumor effect enhancement by immunopathology. PBS, PB, and G-PB were intraperitoneally injected on day 21 after BMT and subcutaneous injection of A20, and the tumor was removed 2 days later. The removed tumor was immunostained with CD3 and NK1.1 to evaluate the transfer of T cells and NK cells to the tumor (a). The number of NK1.1- and CD3-positive cells per field of view was counted for three locations in the tumor (b). ^∗^*p* < 0.01 and^∗∗^*p* < 0.001.

We measured the A20 masses resected from tumor-bearing mice on day 23, 2 days after the 3rd intraperitoneally injection of PBS, PB, or G-PB, to identify cell populations mediating antitumor control induced by stimulation of neutrophils. The A20 masses were compared to control tumors stimulated with PBS to assess the differences in intratumoral CD3^+^ T cell or donor-oriented NK1.1^+^ NK cell infiltrations (Figure 5(a)). No statistical differences in the number of T cells in tumors were detected between the groups (Figure 5(b)). In contrast, the intratumoral infiltration of NK cells was statistically different between the three groups. Although we detected only a few NK cells in the tumors of the PBS group, the number of infiltrated NK cells was significantly increased in the PB group (PBS vs. PB, *p* = 0.004). In addition, the amount of NK cells found in tumors in the G-PB group was significantly higher than the amounts of NK cells in the other groups (PBS vs. G-PB, *p* < 0.001; PB vs. G-PB, *p* = 0.003).

## 4. Discussion

NK cells play an important role in tumor immunosurveillance. For the NK cells to become functional, licensing during the development of NK cells via the interactions between self-specific MHC class I inhibitory receptors-Ly49 in mice and killer cell immunoglobulin-like receptors in humans and self MHC class I molecules is critical [20], and multiple NK cell activating receptors, such as NKG2D and DNAX accessory molecule 1 (DNAM-1), play the complementary or synergistic role [21–23]. Based on our previous report showing that neutrophils functioned as an enhancer of NK cells licensing, we investigated whether the antitumor effects of donor NK cells in recipient mice bearing lymphoma were reinforced by posttransplant *in vivo* neutrophil administration in a murine MHC class I mismatched HSCT model.

First of all, we unexpectedly confirmed that NK cells expressing Ly49G2 receptors corresponding to the recipient-type MHC class I (H-2^d^), not the donor-type MHC class I (H-2^b^), were only transiently activated on day 21 after HSCT. This licensing of NK cells may be caused by the presentation of self MHC class *Ι* derived from recipient nonhematopoietic cells in such lymphoid organs. Barao et al. reported that during reconstitution after HSCT and various activation stimuli, Ly49G2+ NK cells increase and become the “first-responder” cells, which occur independently of NK-cell licensing via Ly49-MHC interactions [12]. However, there was no significant difference between the ratios of Ly49G2^+^ NK cells and Ly49C/I^+^ NK cells in the peripheral blood of posttransplant recipient mice during the experiment period (Figure 1) This might have been affected by the fact that the present HSCT model differed from the mouse combination and transfused hematopoietic cells.

To enhance this licensing of NK cells in recipients, we intraperitoneally injected irradiated PBMNCs, including neutrophils, on day 21 after HSCT. Intraperitoneal administration was used because intravenous injection of PBMNCs was not effective in inducing NK cell licensing, according to our previous report [11]. Additionally, NK cell maturation induced by neutrophils occurs not only in the bone marrow where NK cells develop but also in the periphery where direct NK cells/neutrophil interaction takes place in lymph nodes and the spleen [10]. Reportedly, lactoferrin, elastase, other neutrophil granule-containing proteins, and the direct NK cell-neutrophil interaction occurred through ICAM-3 and CD18 pathways. Moreover, they are also able to induce NK cell activation and cytotoxicity [24–27]. Although the function of irradiated neutrophils remained unclear *in vivo*, it has been reported that radiation can prime neutrophils, rendering them to be more prone to activation [28, 29]. Therefore, irradiated neutrophils could have activated the NK cells independently of licensing *in vivo*.

As a result, licensing of NK cells expressing the Ly49G2 receptor in recipients was enhanced for a short period immediately after injection of PBMNCs and was especially dependent on neutrophil counts. Repeated administration of neutrophil-enriched PBMNCs might be effective in maintaining activated NK cells in recipients.

In the next step, we investigated the effects of licensed NK cells on GVL in tumor-bearing recipient mice after MHC class I mismatched HSCT. We injected A20 cells, a BALB/c-derived lymphoma cell line, subcutaneously into recipient BALB/c mice at the same time as HSCT. Then, irradiated PBMNCs of untreated B10 mice (PB) or rmG-CSF-treated mice (G-PB) were administrated intraperitoneally weekly from day 7 to 21 after HSCT to maintain licensed NK cells in the recipient mice. The irradiated PBMNCs were used to eliminate T cells with strong antitumor effects via the enhancement of the presentation of MHC class I antigens [30]. Significant tumor suppression was observed in the G-PB group compared with tumor suppression in the PB group. Interestingly, the number of donor-oriented NK1.1^+^ NK cells that migrated into the A20 tumors in recipient mice was significantly increased in the G-PB groups (Figure 5(a)), although no differences in the number of T cells infiltrating the tumor lesion were detected among the three groups (PB, G-PB, and PBS). The effects of repeated administrations of irradiated PB or G-PB on acute GVHD in recipient mice were evaluated. The clinically acute GVHD scores were not significantly different among the three groups (Figure 4). Moreover, no differences in T cell migration to the tumor were detected among the PBS, PB, and G-PB groups. These results suggest that the GVL effects in this murine HSCT setting may be enhanced by licensed NK cells expressing the Ly49G2 receptor corresponding to MHC class I (H-2^d^) without exacerbation of acute GVHD. Interestingly, the irradiated neutrophils might have activated the NK cells independently of licensing because donor-oriented Ly49G2^+^ NK cells postdonor engraftment in posttransplant recipient are not licensed by donor-derived neutrophils in the present transplantation setting. In order to clarify the mechanism of this GVL effect, it is necessary either to analyze Ly49 receptors containing Ly49G2 of NK cells infiltrated into the tumor or to eliminate NK cells [12] during injection of neutrophil-enriched PBMNCs in the future studies.

NK cells have diverse activating and inhibitory receptors, and the corresponding ligands provide signals for activation or inhibition of NK cell activity. Most NK cells with inhibitory receptors that encounter self-ligands while interacting with their hematopoietic or nonhematopoietic cells in the MHC class I mismatched HSCT setting are “licensed” to kill the non-self-expressing target. These NK cells can directly mediate GVL effects without GVHD because the NK cells target recipient antigen-presenting cells [3, 31]. Allogeneic NK-cell-mediated GVL effects were demonstrated in murine models of leukemia [3]. Additionally, several acute myeloid leukemia clinical studies demonstrated that NK cells are potent effector cells responsible for the GVL efficacy of HSCT [32–35]. Nevertheless, according to our results, the allogeneic NK-mediated GVL effects were transient after engraftment in the murine MHC class I mismatched HSCT setting. Recently, the effect of NK regulated by neutrophils in human has been reported which found that patients with chronic neutropenia displayed a specific gap in the NK repertoire, associated with poor cytotoxic function and more severe disease manifestations [36]. The GVL effects of NK cells were transient due to a problem at the functional maturation of NK cell after HSCT; thus, appropriate stimulation for NK cell licensing by enriched neutrophils should be needed.

In the clinical setting, G-CSF has been used after transplantation to enhance stem cell engraftment and minimize the morbidity and mortality associated with prolonged neutropenia. The criteria for discontinuing G-CSF treatment after transplant are variable and include the following criteria: on the day of absolute neutrophil count (ANC) > 500/*μ*l × 2 days [37], ANC > 1000/*μ*l [38], or white blood count (WBC) > 1000/*μ*l × 3 days [39]. Therefore, the administration of G-CSF for as long as possible might be useful for activation of NK cell licensing after engraftment. However, we were not able to confirm the enhancement of NK cell licensing by rmG-CSF administration in recipient mice on day 21 after MHC class I mismatched HSCT (data not shown). Therefore, in the setting of human allogeneic HSCT, the regular infusion of irradiated neutrophils isolated from donor peripheral blood into recipients with malignant disease might be effective as adaptive immunotherapy to enhance GVL/GVT effect.

Progression-free survival after HSCT positively correlates with the number of peripheral blood NK cells; thus, the main method to enhance the antitumor effects is expanded NK cell infusion [40, 41]. For NK cell expansion, both *ex vivo* and *in vivo* methods have been developed [42, 43]. In *ex vivo* expansion, methods include single cytokines or cytokine cocktails containing IL-2, IL-15, IL-12, or IL-18 [44, 45]. In addition, anti-CD3 antibodies were added to cultures to potentiate proliferation [46]. Also, feeder cells, such as the chronic myeloid leukemia-derived cell line (K562), in combination with cytokines or expression of membrane-bound IL-15, IL-21, or 4-1BB are often used to enhance NK cell proliferation [47–52]. These methods incur tremendous costs and time and make the use of *ex vivo* NK cell expansion after HSCT less appealing. *In vivo* NK cell expansion has also been extensively investigated. Expansion protocols depend mainly on cytokine infusion, such as IL-2, in recipients [53]; thus, the side effects due to T cell activation are a major drawback of this method. The infusion of irradiated neutrophils derived from the donor after allogeneic HSCT may be a new attractive adoptive immunotherapy that induces *in vivo* licensed NK cell expansion.

To transfer these results to clinically applicable adoptive immunotherapy in patients with malignant disease, further research including the investigation of the NK cells dynamic receptor profile after infusion the neutrophils in allogeneic HSCT is needed. Also, the optimal setting for donor-type neutrophil infusion to enhance NK cell licensing needs to be determined to increase the GVL/GVT effects after allogeneic HSCT.

## 5. Conclusions

Previously, we reported that intraperitoneal neutrophil administration promoted a licensing of NK subsets expressed inhibitory receptors of the Ly49 C-type lectin superfamily through *in vivo* exposure of specific NK receptor ligand using congenic mice without HSCT. In this experiment, we investigated the posttransplant transfer of donor-derived neutrophils in MHC class I mismatched murine HSCT to determine if these cells, independently of licensing, provided enhanced GVL/GVT effects via activation of allogeneic NK cells. Our data indicate that repeated intraperitoneal injections of donor-derived neutrophils after allogeneic HSCT enhance the activity of NK cells licensing via the inhibitory receptors corresponding to the recipient-type MHC class I presented by recipient nonhematopietic cells and induce therapeutic effects in recipient mice bearing lymphoma without exacerbating acute GVHD. Therefore, adoptive immunotherapy with donor-type neutrophils, which enhance NK licensing, is expected to be useful for human HSCT.

## Data Availability

The data that support the findings of this study are available from the corresponding author (SI) upon reasonable request.
